# Patient Portal Use in Diabetes Management: Literature Review

**DOI:** 10.2196/11199

**Published:** 2018-11-06

**Authors:** Ran Sun, Mary T Korytkowski, Susan M Sereika, Melissa I Saul, Dan Li, Lora E Burke

**Affiliations:** 1 Department of Health & Community Systems University of Pittsburgh School of Nursing Pittsburgh, PA United States; 2 Division of Endocrinology and Metabolism University of Pittsburgh School of Medicine Pittsburgh, PA United States; 3 Department of Medicine University of Pittsburgh School of Medicine Pittsburgh, PA United States

**Keywords:** patient portal, diabetes mellitus, personal health records, electronic health records

## Abstract

**Background:**

Health information technology tools (eg, patient portals) have the potential to promote engagement, improve patient-provider communication, and enhance clinical outcomes in the management of chronic disorders such as diabetes mellitus (DM).

**Objectives:**

The aim of this study was to report the findings of a literature review of studies reporting patient portal use by individuals with type 1 or type 2 DM. We examined the association of the patient portal use with DM-related outcomes and identified opportunities for further improvement in DM management.

**Methods:**

Electronic literature search was conducted through PubMed and PsycINFO databases. The keywords used were “patient portal*,” “web portal,” “personal health record,” and “diabetes.” Inclusion criteria included (1) published in the past 10 years, (2) used English language, (3) restricted to age ≥18 years, and (4) available in full text.

**Results:**

This review included 6 randomized controlled trials, 16 observational, 4 qualitative, and 4 mixed-methods studies. The results of these studies revealed that 29% to 46% of patients with DM have registered for a portal account, with 27% to 76% of these patients actually using the portal at least once during the study period. Portal use was associated with the following factors: personal traits (eg, sociodemographics, clinical characteristics, health literacy), technology (eg, functionality, usability), and provider engagement. Inconsistent findings were observed regarding the association of patient portal use with DM-related clinical and psychological outcomes.

**Conclusions:**

Barriers to use of the patient portal were identified among patients and providers. Future investigations into strategies that engage both physicians and patients in use of a patient portal to improve patient outcomes are needed.

## Introduction

### Background

Diabetes mellitus (DM) is a significant public health problem associated with many debilitating health conditions [1]. Prevalence data indicate that approximately 1 of every 10 adults in the United States has diabetes, with predictions that the number will triple by 2050 [2]. The economic burden of diabetes and its complications to the US health care system are enormous. Every 1 in 4 health care dollars is spent for the care of people with diabetes [3]. Thus, the steady increase in the prevalence of diabetes and the substantial associated costs make this one of the most pressing public health concerns in the United States.

Effective diabetes management requires continuous collaboration between individuals and their providers [4], yet the infrastructure of current health delivery systems does not fully support the needs of patients with chronic conditions [5]. A call has been sounded to redesign the care delivery systems to improve chronic disorder care [6]. The Chronic Care Model (CCM) was developed in 1998 to reorganize care delivery to improve functional and clinical outcomes for people with chronic disorders [7]. A primary focus of the CCM is on creating productive interactions between informed patients and a prepared care team [7]. To achieve this, patients need to have the knowledge and skills to make informed decisions, and care teams need to be able to provide relevant patient information, resources, and decision support at the point of encounter. Health information technologies, such as patient portals, can facilitate these activities within health care systems.

Patient portals, often referred to as tethered personal health records (PHRs), provide Web-based platforms for patients’ access to their health information from a health organization’s electronic health record (EHR). Patient portals were widely adopted by health care organizations in the late 1990s and gained greater attention when the Medicare and Medicaid incentive programs for EHR (a.k.a. Meaningful Use) implementation was initiated in 2011 [8]. Today, the PHR adoption rate by consumers is rapidly increasing. It is estimated that the percentage of people who will have a PHR is expected to exceed 75% by 2020 [9]. Patients can perform a variety of medical-related tasks within the portal. For example, most portals permit patients to view laboratory results, receive visit summaries, manage appointments, and electronically communicate with health care providers. More advanced portals enable individuals to record their symptoms and test results, such as blood glucose or blood pressure (BP) readings, data that can be viewed for decision making, and changes in therapy by providers [10]. Health care organizations have commonly adopted patient portals as an essential strategy to provide patient-centered care and engage patients for the purpose of improving clinical outcomes.

### Purpose

Given the continuous increase in the prevalence of diabetes and the increasing development of patient portal applications, a review of the literature on the current use of patient portals in supporting patients with diabetes can be informative. In this review, we identified studies that used qualitative or quantitative methods to describe the state of science in the use of patient portals for diabetes management. Specifically, we evaluated the use of patient portals by patients with diabetes, including the portal functionalities, predictors of portal use, and the effects of portal use on diabetes-related outcomes. These findings provide opportunities for further approaches to improve diabetes management through the use of a patient portal.

## Methods

### Search Strategies

Electronic literature searches were conducted through PubMed and PsycINFO databases. Keywords included “patient portal*,” “web portal,” “personal health record,” and “diabetes.” Additional articles were searched by identifying similar articles in PubMed and manually reviewing the bibliography of published papers in relevant articles. The literature search was limited to publications in the English language and peer-reviewed articles, but no restrictions as to the country in which the study was conducted were imposed.

### Inclusion and Exclusion Criteria

Articles selected were based on the following inclusion criteria: (1) published in the past 10 years (2007-2017), (2) used the English language, (3) study participants were adults (ie, age ≥18 years), and (4) available in full text. Studies using both quantitative and qualitative methods were included in this review. The focus of the selected articles was a patient population of adults with either type 1 diabetes mellitus (T1DM) or type 2 diabetes mellitus (T2DM). Studies were excluded if the portal was designed for parents of children with diabetes.

### Data Extraction

**Figure 1:**
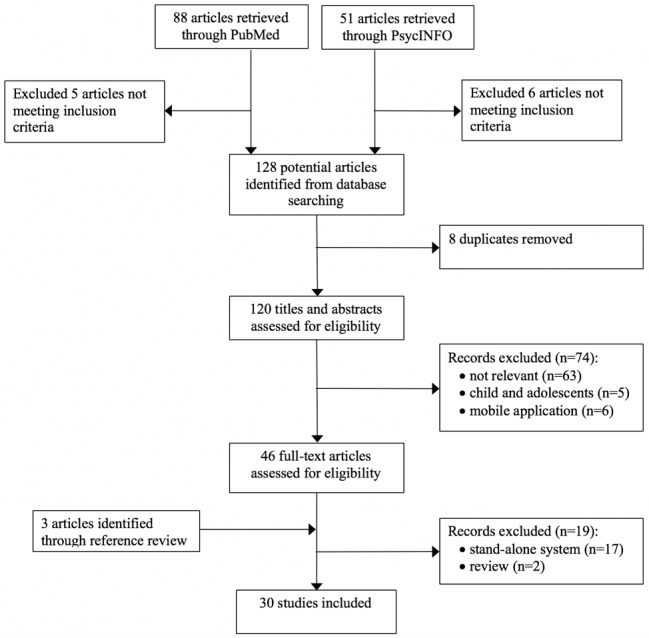
Flow diagram for paper selection process.

The initial search from PubMed and PsycINFO retrieved 128 articles after filtering out 11 articles that did not meet the inclusion criteria. We removed 8 duplicates, which reduced the number to 120 articles for review of the title and abstract. The assessment of these 120 articles resulted in a further removal of 74 articles, including 63 that were not relevant, 5 articles that focused on children, and 6 articles that applied mobile apps for diabetes management. Thus, a review of full text was conducted on 46 articles based on the aforementioned inclusion criteria, and 17 were excluded because of the use of stand-alone Web portals that were not connected to any health care organizations, and, in addition, 2 review papers were excluded. We later added 3 additional articles by searching the bibliography of previously published literature reviews. Therefore, a total of 30 articles were included in our study (see Figure 1), including 6 randomized controlled trials (RCTs), 16 observational studies, 4 qualitative studies, and 4 mixed-methods studies. RCTs and observational studies were summarized based on the following categories: authors and country, study aims and design, sample size and retention, intervention (only for experimental studies), PHR features, measures, and findings. Studies that used qualitative methods or mixed methods were summarized based on study aims, study design, sample, PHR features, measures or questions, and findings (see Tables 1 and 2; Multimedia Appendix 1).

**Table 1:** Randomized controlled trials examining patient portal for diabetes management.

Authors, country	Study aims, design, and level of evidence	Sample and retention	Patient portal features	Intervention	Outcomes (portal related)	Findings
van Vugt et al (2016) [11], Netherlands	2-group study, 6-month randomized controlled trial (RCT) to study the uptake and effects of e-Vita with a self-management support program (SSP) and personalized coaching for patients (Ps) with type 2 diabetes mellitus (T2DM); Evidence: Grade A	N=132; males: 59.1%; white: 91%; age: 67.9 (SD 10.4) years; body mass index (BMI): 30.2 (SD 5.2); glycated hemoglobin (HbA_1c_): 6.6%; retention: Coaching group (CG): 43.9%; noncoaching group (NCG): 59.1%	e-Vita (diabetes mellitus [DM]-specific) by VU University Medical Center allows Ps to access diabetes education; access data from electronic medical records (EMRs) of primary care physicians (PCPs); receive messages from providers; receive SSP	CG (n=66): Personal health record (PHR)+SSP+coaching; NCG (n=66): PHR+SSP	HbA_1c_, BMI, systolic blood pressure (SBP), diastolic blood pressure (DBP), cholesterol, diabetes self-care, diabetes-related distress, and PHR and SSP use	Intention-to-treat (ITT) was applied. PHRs were assessed by 128 Ps, of which 59 Ps never returned to the PHR. The use declined over time. The SSP was used by 5 Ps in the CG and 1 patient in the NCG group, 3 of whom asked a coach for feedback. Ps recently diagnosed actively used the SSP; no differences were observed on outcome measures between baseline (BSL) and 6 months for the 2 groups.
Tang et al (2013) [12], United States	2-group study, 12-month RCT to evaluate an Web-based disease management system by Ps with uncontrolled T2DM; Evidence: Grade A	N=415; Intervention (Int) vs Control (Con): males: 58.9% vs 61%; white: 60% vs 58%; age: 54 (SD 10.7) vs 53.5 (SD 10.2) years; weight: 215.3 (SD 49.4) vs 218.4 (SD 51.3) pounds; HbA_1c_: 9.24 (SD 1.59) vs 9.28 (SD 1.74); Retention: 87%	Web-based diabetes management system (DM specific) by Palo Alto Medical Foundation allows Ps to monitor glucose remotely; view summary report; document nutrition and exercise; record insulin; communicate with the health team; receive advice; personalized education	Int (n=202): access to Web-based disease management system for diabetes; Con (n=213): usual care	HbA_1c_, BP, low-density lipoprotein (LDL), health care utilization, diabetes knowledge, diabetes treatment satisfaction, and depression screening	ITT was applied. Int had reduced HbA_1c_ at 6 months (−1.32% Int vs −0.66 Con, *P*<.001), but not at 12 months. The Int had better LDL control at 12 months (*P*=.001), but no difference for BP or weight. Ps in the Int had a lower distress score (*P*<.001), better knowledge of glucose testing (*P*=.004), better understanding of diabetes (*P*<.001), greater treatment satisfaction (*P*<.001). No differences were noted in the depression screening or health care utilization.
Fonda et al (2009) [13], United States	2-group study, 12-month RCT to examine changes in Problem Areas in Diabetes (PAID), and its association with use of an internet-based diabetes care management (IBCM) program; Evidence: Grade A	N=104; males: 99%; white: 76.7%; age: 60.9 (SD 10.3) years; HbA_1c_: 9.9 (SD 0.9%); Retention not reported	IBCM (DM specific) by VA Boston Healthcare System allows Ps to transmit BP and glucose data from devices; view BP and glucose data; message care managers; access diabetes education	Int (n=52): access to the IBCM program; Con (n=52): usual care	Diabetes distress (PAID), and pattern of usage	The decline in PAID score was significant for sustained users of the portal but not for nonusers in the Int group. Sustained users (n=27) had lower PAID scores at baseline.
McCarrier et al (2009) [14], United States	2-group study, 12-month RCT to test whether a diabetes case management program can improve glycemic control and self-efficacy in adults with T1DM; Evidence: Grade A	N=77; males: 67.5%; white: 96.1%; age: 37.3 (SD 8.09) years; HbA_1c_: 8%; Retention: 83%	Web-based program (DM specific) by University of Washington (UW) General Internal Medicine Clinic allows Ps to view EHR data; upload glucose readings; enter medication, nutrition, and exercise; create action plans; access education	Int (n=41): usual care+Web-based case management program; Con (n=36): usual care	HbA_1c_, diabetes-related self-efficacy, and usage	ITT was applied. A nonsignificant decrease in HbA_1c_ in the Int compared with the Con group (−0.48%, 95% CI −1.22 to 0.27) between groups. The Int group had an increase in self-efficacy compared with the Con group (95% CI 0.01 to 0.59, *P*=.04). The log-in rate was 61%, and averaged 3.3 log-ins per patient. Emails were sent by 44% users, with a mean of 5.0 messages.
Ralston et al (2009) [15], United States	2-group study, 12-month RCT to test Web-based care management of glycemic control using a shared EMR in Ps with T2DM; Evidence: Grade A	N=83; Int vs Con: females: 47.6% vs 51.2%; white: 89.7% vs 73% (*P*=.06); age: 57 vs 57.6; Glycohemoglobin (GHb): 8.2% vs 7.9%; Retention: 89.2%	Web-based diabetes support program (DM specific) by UW General Internal Medicine Clinic allows Ps to access EHR data; communicate with providers; send glucose readings; enter exercise, diet, and medication data; access education	Int (n=42): usual care+Web-based case management program; Con (n=41): usual care	GHb, total cholesterol, SBP, DBP, health care utilization, and usage	ITT was applied. More change in GHb among the Int group compared with the Con group at 12 months (change −0.7%, *P*=.01). SBP, DBP, total cholesterol levels, and use of in-person health care services did not differ between groups. EHR was accessed 76%, 69% emailed, and 33% entered data. Number of page views was not associated with GHb improvement.
Grant et al (2008) [16], United States	2-group study, 12-month RCT to evaluate the impact of a PHR for T2DM; Evidence: Grade A	N=244; Int vs Con: females: 43% vs 56% (*P*=.04); white: 93% vs 84% (*P*=.04); age: 58.8 vs 53.3 years (*P*<.001); HbA_1c_: 7.3% vs 7.4%; Retention: 50.4%	Patient Gateway by Partners Health care system allows Ps to update registration information; send messages; confirm appointments; request prescription refills; access DM modules	Int (n=126): access to a DM-specific PHR (ie, review mediations, and access decision support and care plans); Con (n=118): non-DM-specific PHR	HbA_1c_, BP, and LDL	ITT was applied. More Ps in the Int group had DM treatment adjusted compared with the Con group (53% vs 15%; *P*<.001). There was no difference in HbA_1c_ between groups (Int vs Con: 7.1% vs 7.2%) after 1 year. BP and LDL showed similar patterns at BSL and follow-up between groups.

**Table 2:** Qualitative or mixed methods studies on patient portal for diabetes management.

Authors, country	Study aim	Study design	Sample	Portal features	Measures or questions	Findings
Sieverink et al (2014) [17], Netherlands	To explore factors associated with diffusion of a personal health record (PHR) for patients with type 2 diabetes mellitus (T2DM) in primary health care workers	Semistructured interview with primary care nurses: qualitative	N=11	e-Vita (diabetes mellitus [DM]-specific) by the Diabetes Center in Zwolle allows patients (Ps) to access diabetes education; access electronic health record (EMR) data; receive messages from providers	What are the reasons for using a PHR?; What training do you receive?; How to embed PHR in your daily routine?; What are the barriers and facilitators for embedding PHR in daily routine?; What are your expectations?	Practice nurses indicated barriers for using a PHR: lack of integration with work routines, time constraints, and experience usability problems.
Osborn et al (2013) [18], United States	To understand Ps with T2DM who use MyHealthAtVanderbilt (MHAV) and reasons for use and nonuse, how users are using a portal to manage medications, and explore ideas for functionality improvement	Focus groups and medical chart review: mixed methods	N=75; females: 67%; white: 63%; age: 56.9 (SD 8.8) years	MHAV by Vanderbilt University Medical Center (VUMC) allows Ps to access EHR data; message providers; manage appointments; assess risks; access education	Do you use MHAV or not? How and why?; What could be added to MHAV to help manage medications?; What do you think about an email reminder to refill or dose reminders?	Users were more likely to be white, have higher incomes, and be privately insured. Reasons for nonuse: unaware of the portal (n=3), no access to a computer (n=3), and helped by a family member (n=1). Users used the portal to request prescription refills and view medication list, and Ps were enthusiastic about the idea of adding refill reminder functionality, alerting providers to fill or refill nonadherence, and providing side effects and interactions.
Wade-Vuturo, et al (2013) [19], United States	To explore how Ps with T2DM use and benefit from secure messaging within a patient portal	Focus group and patient survey: mixed methods	N=54; females: 65%; white: 76%; age: 57.1 (SD 8.4) years; body mass index (BMI): 34.4 (10.2); HbA_1c_: 7.0 (SD 1.4)	MHAV by VUMC allows Ps to access EHR data; message providers; manage appointments; assess risks; access education	HbA_1c_, self-reported frequency of use, benefits and barriers to use messaging	Greater use of messaging to schedule an appointment was associated with patients’ glycemic control (*r*=−.29, *P*=.04). Benefits of messaging: improved patient satisfaction, enhanced efficiency and quality of face-to-face visits, and access to care. Barriers to use messaging: negative experiences with messaging. Ps’ assumptions about providers’ opinion and instruction.
Urowitz et al (2012) [20], Canada	To evaluate the experience of Ps with T1DM or T2DM and providers using a Web-based diabetes management portal	Telephone interview and open-ended questionnaire: qualitative	Ps (n=17); females: 53%; providers (n=64)	Patient portal by the Waterloo Wellington Local Health Integration Network allows Ps to access DM education; access EHR data	Telephone interview with Ps and open-ended questionnaires with providers	17 Ps were interviewed. Facilitators of disease management: increase awareness of their disease, access to educational information, and promote behavior change. Barriers to portal use: poor usability, not useful, challenges with physician engagement, and lack of understanding. Recommendations for portal improvements: more Web-based tutorial about the portal content, improve usability.
Mayberry et al (2011) [21], United States	To examine the role of health literacy, numeracy, and computer literacy on usage of a patient Web portal (PWP) in Ps with T2DM	Focus group and patient survey: mixed methods	N=75; females: 68%; white: 47%; age: 56.9 (SD 8.8) years	MHAV by VUMC allows Ps to access DM education; access EHR data	Health literacy, numeracy, computer literacy, self-report usage of PWP and health information technology (HIT)	Lower health literacy was associated with less use of a computer for searching diabetes medications or treatments, but not usage of a PWP. Numeracy and computer literacy were not associated with PWP use. Family members’ support facilitated Ps usage of both PWP.
Bryce et al (2008) [22], United States	To rate the potential or actual usefulness of 15 features of a Web-based portal for diabetes management	Focus group and patient survey: mixed methods	Preportal group (n=21) vs portal-user group (n=18): nonwhite: 33% vs 22%; age: 53 (SD 13) vs 55 (SD 11) years	HealthTrak by University of Pittsburgh Medical Center (UPMC) allows Ps to access EMR data; schedule appointments; message providers; access education; logbooks	The study asked how the portal affected management of diabetes, Ps’ experiences in using the portal and communicating with physicians	Features rated most favorably were: calculator to estimate blood glucose control (74%), appointment reminder (74%), email to health team (74%), personal tracking logs (69%), and scheduling (69%). More patients from the preportal group than the portal-users group favored personal logs (*P*=.02) and opportunities to form interest groups (*P*=.03).
Zickmund et al (2008) [23], United States	To examine the impact of the provider-patient relationship on interest in using the patient portal	Focus group: qualitative	N=39; white: 72%; males: 52%; age: 54 (SD 12)	HealthTrak by UPMC allows Ps to access EMR data; schedule appointments; message providers; access education; logbooks	Topics included the relationships with providers, and feedback on the patient portal	Interest in the portal was linked to dissatisfaction with provider responsiveness, unable to obtain medical information, and logistical problems. Disinterest in the portal was linked to satisfaction with the provider communication, difficulty in using the portal, and fear of losing connections with providers. No patient identified email communication through the portal was helpful
Hess et al (2007) [24], United States	To assess the impact of HealthTrak on patient-provider communication during September 2004-January 2007	Focus groups: qualitative	N=39; males: 51%; white: 72%; age: 54 (SD 12) years	HealthTrak by UPMC allows Ps to access EMR data; schedule appointments; message providers; access education; logbooks	Discussion around living with diabetes, desired information about diabetes, current sources of information about diabetes, doctor-patient communication, and reaction to the portal	The number of patient visits or telephone calls received did not change, but the number of HealthTrak messages increased. Participants felt that the system enhanced communication. Having access to laboratory tests was preferred. They became frustrated when test results were not released, or messages were not answered by providers.

### Quality Assessment

The quality of the reviewed studies that used quantitative methods was assessed using the evidence grading system developed by the American Diabetes Association. An evidence grade of A, B, C, or E is assigned depending on the quality of the evidence. A grade A evidence is considered optimal because it is derived from large, well-designed clinical trials or meta-analyses; it is estimated to have the best chance to improve outcomes when applying the treatment to the appropriate population. Grade B ratings indicate supporting evidence from well-conducted cohort studies or case-control studies. Grade C ratings indicate supporting evidence from poorly controlled or uncontrolled studies. A separate category E is applied to papers reporting expert opinions or clinical experience when there is no evidence from clinical trials.

## Results

### Description of Included Studies

We reviewed 30 studies focusing on 13 different portals from 3 countries—10 from the United States, 2 from the Netherlands, and 1 from Canada. Of these 13 portals, 5 were designed for patients with diabetes and functioned as a component in Web-based diabetes management programs. These 5 DM-specific patient portals were from the Palo Alto Medical Foundation, VA Boston Healthcare System, University of Washington General Internal Medicine Clinic, the VU University Medical Center, and the Diamuraal of the Netherlands. Almost half of the included studies (n=13) focused on patients with T2DM, 1 on patients with T1DM, 6 included both types, and 10 did not specify.

Of all the studies included, 6 [11-16] were RCTs (Table 1). These studies examined the effect of a DM-specific patient portal on diabetes-related outcomes. The sample sizes for the RCTs ranged from 77 to 415, with the number of subjects in 2 studies being less than 100 [14,15] and in 1 study more than 400 [12]. The study duration in the 5 RCTs was 12 months [12-16], with the duration of the remaining RCTs being 6 months [11]. Of 6 RCTs, 5 reported a retention rate range of 50.4% to 89.2% and employed an intention-to-treat approach to handle protocol deviations [11,12,14-16]. These 6 RCTs studied an array of diabetes-related outcomes, including glycated hemoglobin (HbA_1c_) or glycohemoglobin (GHb), systolic blood pressure (SBP) and diastolic blood pressure (DBP), body mass index (BMI reported as kg/m^2^), total cholesterol, and low-density lipoprotein (LDL). The psychological outcomes that were examined included diabetes-related distress and diabetes-related self-efficacy.

There were 16 observational studies [25-40] identified, which included 3 retrospective cohort studies [25,29,38] and 13 cross-sectional studies (Multimedia Appendix 1). The sample sizes of these studies were variable; 7 studies [25,29,31,33,35,37,39] had more than 10,000 participants, and 5 studies [26,30,34,36,40] had less than 1000. The data only obtained from the EHR were examined in 7 studies [25,29,30,33,34,38,39], and 9 studies [26-28,31,32,35-37,40] combined data collected from the EHR and patient surveys. The association between patient portal use and diabetes-related outcomes was investigated in 5 studies; 1 of the studies examined the overall portal use [33], whereas the other 4 studies investigated only certain features within the portal, such as secure messaging [25,39,41] or medication refills [25,29]. The remaining 11 studies examined the usage of the patient portal and factors associated with portal use [26-28,30-32,35-38,40].

Qualitative methods were used in 4 studies [17,20,23,24], and 4 additional studies used mixed methods [18,19,21,22] to address the benefits and barriers of using patient portals (Table 2). Focus group was used in 6 studies [18,19,22,23,42,43], of which 4 [18,19,22,42] also used patient surveys. The sample sizes in the 6 studies using focus groups ranged from 39 to 75 [18,19,21-24]. In 1 study, semistructured interviews with 11 primary care nurses were conducted [30]. Another study conducted telephone interviews with 17 patients and collected qualitative data using open-ended questionnaires from 64 providers [20].

### Features Provided in Patient Portals

Features offered in patient portals varied across systems. Most portals allowed patients to access a component of the EHR data (eg, visit summary, medical history, physical examination results, lab results), receive general health education, request prescription refills, and communicate with health care providers. In the DM-specific portals, patients were able to perform more activities such as wirelessly uploading their blood glucose readings assessed via home-monitoring devices [12-15,26]. The education provided in these DM-specific portals was specifically related to patients’ conditions and prescribed medications [12-16]. A few portals also enabled patients to enter lifestyle data such as diet and exercise [12,14,15,25]. In 4 RCTs, the interventions included access to the portal and assigned case managers (nurses, dietitians, or pharmacists) to assist patients in using the Web-based portal, responding to messages, reviewing blood glucose levels and food intake, and adjusting medications as appropriate [12-15].

### Patient Usage of the Portals

The percentage of patients with diabetes who registered for a portal account ranged from 29% to 46% [28,30,37,39]. Among patients with portal accounts, 27% to 76% actually logged on to the portal at least once [13,27,28,30,35,37]. However, 50% (3/6) of these studies indicated a response rate of less than 50% [27,28,30]. In 2 studies, an initial high log-in frequency was observed that declined over time [11,30]

Patients logged on to portals for various tasks. Of all included studies, 1 study identified viewing laboratory results as the most frequently used feature, followed by requests for medication refills, sending and reading messages, and making appointments [35]. Another study reported similar findings, with checking which laboratory tests were ordered by providers being the most frequent activity, followed by reading messages from providers and reviewing laboratory results [33].

### Patient Characteristics of Portal Users and Nonusers

Significant differences between portal users and nonusers have been identified. Portal users were more likely to be younger [25,27,32,33,35,38], white [18,25,33,35], and male [25,32,38] with higher incomes [18,33,38] and greater educational attainment [27,32,33,35]. Other factors reported to be associated with portal use were higher health literacy [37] and higher morbidity [38]. Ronda et al found that insulin use, T1DM, longer duration of diabetes, polypharmacy, and treatment by an internist were associated with using the portal [26,27,32].

### Impact of Patient Portals on Glycemic Control

The impact of DM-specific patient portals on glycemic control was investigated in 5 RCTs. Of these, 4 targeted patients with T2DM and yielded inconsistent results. Tang et al randomized 415 patients to either the usual care group or the intervention group. The results demonstrated reductions in HbA_1c_ in the intervention group, where patients had access to a Web-based diabetes management system, compared with that of the usual care group (−1.32% vs −0.66%, *P*<.001) at 6 months, but the difference between groups was no longer significant at 12 months (−1.14 vs −0.95%, *P*=.13) [12]. Ralston et al observed that the intervention group (n=42) in which patients were introduced to the Web-based diabetes support program had a greater decline in GHb than the usual care group (n=41) at 12 months (difference in mean change between groups=−0.7%, *P*=.01) [15]. Another 2 RCTs provided patients with access to portals in both groups. The only difference between groups in the study conducted by Grant et al was the content of the module that was diabetes related in the intervention group but not the control group [16]. In the study by Vugt et al, patients in the intervention group, but not in the control group, were able to request feedback from a health coach [11]. Both these studies failed to observe changes in HbA_1c_ over time in either group [11,16]. The study by McCarrier et al, which examined 77 patients with T1DM, did not find a significant decrease in the average HbA_1c_ in the intervention group with a Web-based management program when compared with the usual care group over 12 months [14].

There were 3 observational studies that used data from EHR as well as an audit of portal registration and usage to examine the association of portal use with glycemic control. Of these 3 studies, 2 studies focused on single features (ie, secure messaging, Web-based medication refill). The 5-year retrospective cohort study conducted by Shimada et al in 111,686 veterans demonstrated that patients with HbA_1c_ ≥7% at baseline tended to achieve HbA_1c_ <7% with 2 (odds ratio [OR] 1.24, 95% CI 1.14 to 1.34) or more (OR 1.28, 95% CI 1.12 to 1.45) years of messaging use. Use of Web-based medication refills was not associated with changes in glycemic control [25]. An earlier study of 15,427 patients that examined the messaging feature revealed that frequent use of messaging (ie, ≥12 threads) was associated with HbA_1c_ less than 7% (relative risk [RR] 1.36, 95% CI 1.16 to 1.58) [39]. Another study of 10,746 adults, which investigated the association between overall portal use and diabetes quality measures, observed a minimum decrease in HbA_1c_ was associated with an increase in portal use (0.02%, *P*<.01) [33].

### Impact of Patient Portals on Other Diabetes-Related Outcomes

In addition to glycemic control, researchers also explored other diabetes-related physiological outcomes. The RCT by Tang et al found that patients who had Web-based access to the diabetes management system had better control of LDL, but not BP or weight, when compared with patients in the usual care group at 12 months (*P*=.001) [12]. A significant decline in LDL and BP was observed in 2 retrospective cohort studies that examined single features in the portal [25,29]. Sarkar et al focused on individuals with diabetes who were prescribed statins. They observed that for patients with poor adherence to a statin medication at baseline (n=3887), those who requested all their medication refills on the Web during the 5-year study period had a 2.1 mg/dL decrease in LDL compared with nonusers (95% CI −4.4 to 0.18). This decrease in LDL can be explained by the improved statin adherence [29]. Shimada et al demonstrated that both secure messaging use and Web-based medication refill requests were associated with lower LDL at follow-up. Patients with uncontrolled BP at baseline tended to achieve better control at follow-up, if they used the Web-based medication refill function for 2 (OR 1.07, 95% CI 1.01 to 1.13) or more years (OR 1.08, 95% CI 1.02 to 1.14) [25]. Significant associations between portal use and improved physiological measures were reported by 2 other cross-sectional studies [33,39]. Tenforde et al reported that portal users (n=4036), compared with nonusers (n=6170), had a small difference in SBP (by 1.13 mm Hg, *P*<.01) and DBP (by 0.54 mm Hg, *P*<.01) [33]. In the Harris et al study of 15,427 patients, a small but significant association was observed between secure messaging use and LDL <100 mg/dL (*P*<.001) [39]. Other studies did not find a difference in total cholesterol [11,15], LDL [15,16,33], BP [11,12,15,16,39], or BMI [11] between groups.

Several studies also assessed changes in psychological measures, including diabetes-related distress and self-efficacy for managing diabetes. Data on diabetes-related distress as measured by the Problem Areas in Diabetes (PAID) questionnaire were reported in 4 studies. Of these studies, 1 study using an RCT design found a lower distress score in the intervention group (n=202) compared with the usual care group (n=213, 0.6, SD 0.8, vs 1.0, SD 1.0, *P*<.001) at 12 months [12]. No significant differences were found between treatment groups in the PAID scores in 3 other studies, including 2 RCTs [10,12] and 1 observational study [31].

Self-efficacy between groups was assessed in 2 studies. In an RCT by McCarrier et al (n=77 patients with T1DM), the intervention group had a significant increase in diabetes-related self-efficacy compared with the control group (*P*=.04) [14]. The study from the Netherlands analyzed data from 1390 respondents and found a significantly higher self-efficacy score for portal users (ie, patients with at least 1 log-in, 79.5, SD 15.8) than nonusers (ie, patients without a log-in, 72.7, SD 17.8) among patients with T2DM (n=1262, *P*<.001) but not T1DM (n=128) [32].

### Qualitative Studies Reporting Benefits and Barriers to Using Patient Portals

There were 8 studies that evaluated patient portals by applying qualitative methods—6 used focus groups, 1 used face-to-face interviews, and 1 used telephone interviews. Qualitative responses revealed that patients favored features that allowed them to view summaries, request prescription refills, receive reminders for medical appointments, access laboratory results, and communicate with providers [18,22,24]. Patients stated that benefits of using the portal included more awareness of their disease, increased access to care outside of office visits, enhanced communication and satisfaction, and promotion of behavior change [19,20,24].

Patients who never used the portal provided the following reasons for not requesting a log-in: unawareness of the existence of the portal, no use of computers, family members as delegates, slow response from physicians or nurses, and poor usability of the portal [18,20,24]. Mayberry et al highlighted the role of family members in supporting patients’ access to and use of the portal, especially for those with limited health literacy, numeracy, or computer literacy. Family members taught the patient how to use each function in the portal, and some acted as delegates for patients by managing their health conditions [21]. Several studies also identified that physician engagement in using the portal remains challenging. Providers with negative attitudes toward the portal listed lack of integration with work routine, minimal knowledge about the portal, limited time, and usability problems as reasons for not using the portal [20,30].

## Discussion

### Principal Findings

This literature review reports on the current evidence on EHR portal use in the clinical management of patients with diabetes. The 13 patient portals that were represented in the 30 studies showed wide variability in features examined and provided across portals, evaluated diabetes outcomes, and whether the technology resources were applied in combination with a disease management program for diabetes. These variabilities increased the difficulty of performing a meta-analysis and generating any conclusions about the effectiveness of patient portals for diabetes management. In our review of the RCTs, we found inconsistent findings regarding the effect of the portal use on diabetes outcomes. Observational correlational studies also yielded mixed findings regarding the association between portal use and diabetes outcomes. However, we were able to identify that the patient portal, which leverages strong patient-centered principles (eg, DM education, tailored feedback on patient’s DM-related health data), performed better in improving patient outcomes. The DM-specific portals enabled patients to receive personalized education, send blood glucose readings, and obtain individualized feedback from the health team.

Although we observed more favorable outcomes associated with using the DM-specific portals, the effect sizes in the studies reviewed were small. This may be due to several challenges associated with the use of patient portals. The design of the majority of the patient portals currently available was not patient-centered, meaning that features provided do not align with patient expectations, and in many cases were not evidence based. For a self-management intervention to be effective, appropriate theories of engagement and implementation should be in place to support the evidence-based intervention. For example, to ensure the effective application of a system, the system needs to provide a complete feedback loop, which consists of multiple components that include monitoring and transmission of patient status, data interpretation in comparison with personalized goals, adjustment of treatment regimen based on patient status, timely communication with individualized recommendations, and repetitiveness of this cycle [44]. However, from the studies reviewed, current patient portals often provided only one of these functions or a subset of them, which may contribute to the less robust favorable results. To significantly improve diabetes management, patient portals need to do more than provide convenient services such as requesting medication refills or reviewing laboratory results. They should also integrate more evidence-based strategies, such as patient education, to enhance patient engagement.

The current state of low engagement by patients in portal use may interfere with the ability to achieve meaningful clinical benefits. Initial high log-in rates followed by a rapid decline in portal use suggest that multifaceted barriers prevent patients from engaging in the long-term use of patient portals. These barriers are technology-related (eg, functionality, usability), patient-related (eg, access to the internet or a computer, low health literacy, perceived usefulness, sociodemographic and clinical characteristics), and provider-related (eg, provider engagement).

A recently published review indicated that endorsement from providers was one of the most influential factors that contributed to patients’ accepting the portal and using it as a tool for diabetes self-management [8]. However, health care providers commonly expressed concerns toward using a patient portal such as a disruption of their workflow and time constraints. These challenges may limit physicians’ adoption and engagement of portal use and lead to minimal improvement in patient outcomes [45]. Future research needs to focus on addressing these barriers to promote more physician involvement in using the portal.

### Limitations

There were several noted limitations of this review. First, our findings lacked sufficient quality evidence; the results of this review are not well-supported by level A evidence, with the majority of studies graded as the B or C level. It is no longer feasible to randomly assign patients to either portal use or nonuse group as individuals have the right to access their health information, but studies could consider examining different designs or additional features, given the necessary health information included in the portal. Second, this literature review only included studies explicitly concerned with patient portals and diabetes, studies evaluating patient portals for multiple chronic disease management that may include diabetes were not included. Finally, only 1 person was involved in the selection of the studies for inclusion in our review. Future studies should consider using a multiple-rater approach for study evaluation and data extraction.

### Conclusions

In conclusion, this review identified several opportunities that could potentially improve diabetes outcomes through a patient portal. Because the majority of the studies examined the overall effect of patient portals, future investigations should consider investigating single features to understand the contribution of each component and understand which component is more influential than others in helping patients manage their diabetes. Moreover, a conceptual framework is needed to standardize an approach to guide the design and evaluation of patient portals. Specifically, functionalities need to be specified to provide guidance on system requirements for patient portal developers. Moreover, a set of evaluation metrics needs to be developed for the evaluation of patient portals to enable them to be compared and ranked. To further improve diabetes outcomes, continued investigation of strategies that could potentially enhance the implementation of the patient portal (eg, portal design, implementation strategy) may enable the patient portal to reach its fullest potential in supporting diabetes management and increasing patient engagement. At the same time, physicians’ perceptions of portal use need to be assessed, and potential barriers need to be addressed to foster physicians’ engagement in patient portals.

## Appendix

Multimedia Appendix 1 Observational studies examining patient portals for diabetes management.

## Abbreviations

BMI: body mass index
BP: blood pressure
CCM: chronic care model
DBP: diastolic blood pressure
DM: diabetes mellitus
EMR: electronic medical record
EHR: electronic health record
GHb: glycohemoglobin
HbA_1c_: glycated hemoglobin
LDL: low-density lipoprotein
PAID: Problem Areas in Diabetes
PHR: personal health record
RCT: randomized controlled trial
SBP: systolic blood pressure
