# Supplementary material for: Patient Portal Use in Diabetes Management: Literature Review
Source: JMIR Diabetes. 2018 Nov 6;3(4):e11199. doi: 10.2196/11199 (PMC6246970; doi:10.2196/11199)
Supplement: Multimedia Appendix 1 [file diabetes_v3i4e11199_app1.pdf]

Multimedia Appendix 1. Observational Studies Examining Patient Portals for Diabetes Management

Authors and country	Study aims, design, and level of evidence	Sample	Portal features	Outcomes (portal-related)	Results
Shimada et al (2016), US [25]	5-year retrospective cohort study to examine the association of secure messaging (SM) use and Web-based prescription refills use with physiological measures among Patients (Ps) with type 2 diabetes mellitus (T2DM)<br><br>Evidence: Grade B	N=111,686; user 45.2%; females: 3.6%; white: 68.9%; age 62.1 (SD 9.6) years	My Healthe*e*Vet by Department of Veterans' Affairs allows Ps to:<br>1) enter data for diet, activity, and vital signs<br>2) set goals<br>3) access data from electronic health record (EHR)<br>4) communicate with providers<br>5) request prescription refills	HbA1c, low-density lipoprotein (LDL), blood pressure (BP), SM use, and prescription refill use	34.1% of the sample used refill and 15.8% used SM. Users were younger (*P*<.001), and more likely to be female (*P*<.001). Ps with uncontrolled glucose were more likely to achieve glycemic control after ≥2 year of SM use. Ps with uncontrolled BP were more likely to achieve BP control after ≥2 years of refill use. No association of refill use with glycemic control were noted. Both features were associated with lower LDL at follow-up.
Ronda et al (2015), Netherlands [26]	Survey study of Ps with diabetes mellitus (DM) to understand their experiences with a web portal<br><br>Evidence: Grade C	N=632; T2DM: 81.8%; males: 63.1%; white: 92.8%; age 59.7 (SD 13.2) years; HbA1c: 7.2%	Digitaal Logboek (diabetes mellitus [DM]-specific) by Diamuraal allows Ps to:<br>1) access data from EHR<br>2) review medication list<br>3) receive diabetes education<br>4) view examinations and a visit summary<br>4) upload glucose remotely<br>5) message providers	login frequency, perceived usefulness, and diabetes knowledge	Insulin use (odds ratio [OR] 2.07), frequently experiencing hyperglycemic episodes (OR 1.30), and better diabetes knowledge (OR 1.02) increases the odds of being a persistent user (≥2 times). Early quitters (n=219) felt items were not applicable to their situation. Ps prefer a reminder function and including medication information and side-effects.
Ronda et al (2014), Netherlands [27]	Survey study of Ps with DM on their opinions and the barriers to requesting a login and to using a portal<br><br>Evidence: Grade C	N=1,390; T2DM: 77.5%; regular use (n=632) vs. nonuser (n=758); males: 62.7% vs. 56.5%; Caucasian: 93.6% vs. 89.3%; age: 60.2 vs. 68.1 years; HbA1c: 7.1% vs. 6.8%	Digitaal Logboek (DM-specific) by Diamuraal allows patients to: same as above	self-reported usage, reasons for requesting or not requesting a login, how they heard of the portal, frequency and duration of portal use, who helped add data to the portal	14% were nonusers among Ps with type 1 diabetes mellitus (T1DM) compared to 69.2% among Ps with T2DM.<br>The main reason for not using was unawareness (72.4%). Younger age, higher education, being treated by an internist, insulin use, polypharmacy, better diabetes knowledge, and more hyperglycemic episodes were associated with portal use. Nonusers perceived specific portal content as less useful.

Roelofsen et al (2014), Netherlands [28]	Cross-sectional study to explore the differences in Ps with T2DM who were interested and uninterested in the portal<br><br>Evidence: Grade C	N=1,378; Interested (n=974) vs uninterested (n=404); males: 56.6% vs. 48.5%; age: 62.3 (SD 9.7) vs. 68.4 (SD 9.7) years; body mass index (BMI): 29.8 (SD 5.0) vs. 30.2 (SD 5.5)	e-Vita (DM-specific) by the Diabetes Center in Zwolle allows Ps to:<br>1) receive messages<br>2) review checkup results<br>3) set goals and actions<br>4) monitor metabolic values<br>5) receive education	Usage	Of the 974 Ps who interested in the portal, 405 (41.6%) were registered for it, and 110 (27.2%) actually logged on to the portal. Interest Ps were more likely to be male, younger, higher educated, and have shorter T2DM duration.
Sarkar et al (2014), US [29]	Observation cohort study to determine the statin adherence before and after using the refill function in the portal between 2006 and 2010<br><br>Evidence: Grade B	N= 17,760 males: 54%; white: 58%; age: 62.7 (SD 11) years	Web-based portal kp.org by Kaiser Permanente Northern California (KPNC) allows Ps to:<br>1) request medication refills<br>2) view medical history and office visit summary<br>3) view laboratory results<br>4) schedule appointments<br>5) message providers	Statin adherence, LDL, use of refill function	49% (n=8,705) of the cohort used the refill. LDL decreased by 3.1 mg/dL among exclusive users (i.e. request all statin refills on the Web) than nonusers. Nonadherence declined by 6% (95% confidence interval [CI]: 4%–7%) among exclusive users, without changes among occasional users (request refills on the Web as least once). No differences were identified between occasional users and non-users. The improvement in LDL was partially mediated by improved adherence.
Sieverink et al (2014), Netherlands [30]	Descriptive study to understand the usage pattern of the first 6 weeks using the personal health record (PHR) by patients with T2DM<br><br>Evidence: Grade C	N=568	e-Vita (DM-specific) by the Diabetes Center in Zwolle allows Ps to: same as above	Number of logins, time and day of the action, actions taken, information reviewed, and goals added	28% of all registered users (n=161) visited e-Vita at least once in the first 6 weeks, the number declined over the weeks. 93% of users ended their session the first time they visited the education session.

Lyles CR, et al (2013), US [31]	Survey design to examine the association between patient-provider communication or trust ratings and 1) being a registered user and 2) use of SM<br><br>Evidence: Grade C	N=14,102; males: 51%; white: 33%; age≥60 years: 50%	Web-based portal by KPNC allows Ps to: same as above	Portal use in the 2 years during or following survey completion (2006–2007), secure message use, communication, trust	36% of the user used messaging. Increased trust was associated with being a registered user among white, Latino, and older Ps, as well as SM use among white Ps. Better communication ratings were related to being a registered user.
Ronda et al (2013), Netherlands [32]	Survey study design to examine the differences of Ps with and without a login by DM type<br><br>Evidence: Grade C	N=1,390; T1DM: 9.2%; males: 59.4%; age: 63.9 (SD 12.2) years; response rate: 67%	Digitaal Logboek (DM-specific) by Diamuraal allows patients to:<br>1) access data from EHR<br>2) receive general diabetes education<br>3) view all examinations and diabetes visits<br>4) upload glucose level remotely<br>5) contact with care provider	Diabetes treatment satisfaction, diabetes-specific distress, general well-being, diabetes management self-efficacy, and diabetes knowledge	Among 128 Ps with T1DM, those with a login (89.8%) were younger, had better diabetes knowledge, and treated by an internist. In 1,262 Ps with T2MD, fewer Ps had a log-in (41.0%), and having a login was associated with younger age, male, higher education, treatment by an internist, longer diabetes duration, and polypharmacy (all *P*<.001). Ps with a login perceived more diabetes-related distress, more hyper- and hypo- glycemic episodes, more self-efficacy, and better diabetes knowledge.
Tenforde et al (2011), US [33]	Retrospective audit of PHR use during July 2008–June 2009 to measure the association between PHR use and diabetes quality measures<br><br>Evidence: Grade C	N=10,746; user vs nonuser; females: 46% vs. 50% (*P*<.01); white: 84% vs. 66% (*P*<.01); age: 59 (SD 10) vs. 62 (SD 10) years (*P*<.01) Income: 53,000 vs. 47,500 (*P*<.01)	MyChart by Cleveland Clinic allows Ps to:<br>1) access data from electronic medical record (EMR)<br>2) view glucometer readings<br>3) access diabetes education<br>4) receive reminders for diabetes-related tests<br>5) communicate with providers	HbA1C, LDL, BP, BMI, ACEi/ARB use and/or microalbumin testing, pneumococcal vaccination, foot and dilated eye examination, and smoking status, PHR use	Compared to non-users (n=6,710), PHR users (n=4,036) were younger, had higher income and education, tend to be Caucasian. PHR users had lower HbA1c (by 0.29%), SBP (by 1.13 mmHg), and DBP (by 0.54 mmHg) (all *P*<.01). An incremental increase in PHR use days by 10 was associated with greater odds of having decreased HbA1c values (0.02%, *P*<.01).

Bredfeldt et al (2011), US [34]	Retrospective study to determine whether interaction with physician between office visits provide better care during January 2007–December 2008<br><br>Evidence: Grade C	N=174 primary care physician (PCPs); Ps panels that are white or mixed race vs. black or Hispanic:<br>age:<br>57.8 (SD 2.3) vs. 57.9 (SD 2.5) years;<br>Income:<br>$90,359 vs. $60,499	MyChart by Kaiser Permanente, Mid-Atlantic States allows Ps to:<br>1) access laboratory and pharmacy information<br>2) schedule appointments<br>3) communicate with physicians	Diabetes Recognition Program (DRP) score, use of messaging and number of phone calls	Physicians (n=116) whose Ps were white or mixed race tend to use more messaging and phone with their Ps between visits. No association between such contacts and DRP scores was noted. Physicians (n=58) with black or Hispanic Ps had higher DRP scores associated with the messaging (*P*<.01).
Sarkar et al (2011), US [35]	Survey method to examine Ps use patterns of the kp.org by patients with DM during January–December 2006<br><br>Evidence: Grade C	N=14,102; females: 49%; non-white: 78%; age: 50-59 33%; HbA1c: 7.59%;	kp.org by KPNC allows Ps to:<br>1) view lab results<br>2) communicate with providers<br>3) request medication refills<br>4) schedule appointment	Proportion of Ps who activated accounts, logged on, and use of health-services functions	40% of the 14,102 Ps requested a password for the portal. Of these, 4311 (76%) activated the accounts, and 69% logged on; 53% viewed laboratory results, 38% requested medication refills, 37% sent messages, and 15% made appointments. African-Americans and Latinos had higher odds of never logging on (OR 2.6; OR 2.3) compared to non-Hispanic Caucasians, as did those without an educational degree (OR 2.3).
Cho et al (2010), US [36]	Cross-sectional survey to measure veterans' access to and use of the Internet, and their interest in using the portal for T2DM<br><br>Evidence: Grade C	N=201; males: 97%; white: 60%; age: 58.9 (SD 10.4); HbA1c: 9.6%	My Health*e*Vet by Department of Veteran Affairs Medical Center allows Ps to:<br>1) access EHR data<br>2) enter medications, glucose and BP readings<br>3) request prescription refills<br>4) access provider notes<br>5) receive reminders<br>6) message providers	Awareness and current use of the Web portal, and interest in using it to manage diabetes	41% are very interested in using MHV to track blood glucose readings at home. A third did not have access to internet at home. Factors associated with being very interested were: having internet access at home (*P*<.001), "a lot/some" trust in the Internet as a source of health information (*P*=.002), younger age (*P*=.03), and some college (*P*=.04).

Sarkar et al (2010), US [37]	Survey study design to investigate use of an internet-based patient portal among adults with DM during January–December 2006<br><br>Evidence: Grade C	N=14,102; females: 49%; non-white: 78%; age: 50-59 33%; HbA1c: 7.59%	Patient portal by KPNC allows Ps to: same as above	Health literacy, use of each feature in the portal	40% (n=5,671) registered, 76% (n=4,311) logged in. The pages visited were view laboratory results, request medication refills, send messages to providers, and make appointments. People with limited health literacy had higher odds of never signing on to the portal (OR 1.7, 95% CI 1.4–1.9).
Weppner et al (2010), US [38]	Retrospective cohort study to describe use of a web-based shared medical record (SMR, MyGroupHealth) by older patients with DM during August 2003–August 2007<br><br>Evidence: Grade B	N=6,185; females: 50.9%; age: 75.2 (SD 6.7) years	MyGroupHealth by Group Health Cooperative allows Ps to:<br>1) view EHR data<br>2) request medication refills<br>3) make appointments<br>4) communicate with providers	Initial use, subsequent use, PCP's use of the communication feature	32.2% (n=1,990) used the SMR. Portal use was associated with younger age, male, and higher socioeconomic status, overall morbidity, and PCP's use. SMR use was more likely within 3 months of an increase in morbidity (hazard ratio 1.61, 95% CI 1.28–2.01) and within 1 month of changing to a PCP with higher SM use (hazard ratio 3.02, 95% CI 1.66–5.51).
Harris et al (2009), US [39]	Cross-sectional analysis to test the association of electronic messaging with care quality for DM or outpatient utilization between January 2004–March 2005<br><br>Evidence: Grade C	N=15,427	MyGroupHealth by Group Health Cooperative allows Ps to: same as above	HbA1c, BP, LDL, outpatient visits, use of electronic messaging	34% (n=5,274) registered the portal, and 19% of Ps (n=2,924) used electronic messaging. Frequent use of electronic messaging (i.e.≥12 threads) was associated with A1C<7% (RR 1.36, 95% CI 1.16–1.58), a higher rate of outpatient visits (RR 1.39, 95% CI 1.26–1.53), but not BP. Small but significant association was observed between secure messaging and LDL<100 mg/dl.
Wald et al (2009), US [40]	Survey design to examine patient journal use and patient experience using the diabetes journal by Ps with T2DM during 2005–2007<br><br>Evidence: Grade C	N=126; males: 58%; white: 93%; age: 59.4 years; HbA1c<7.0%: 60%; response rate: 67%	Patient Gateway by Partners Health care allows Ps to:<br>1) access data from EHR<br>3) enter concerns and requests about glucose, cholesterol, and BP control<br>4) request for referrals or education<br>5) change medication and allergy list	Use of the electronic journal, journal experience	A diabetes care plan took 5–9 minutes to complete by the patient. 61% reported they talked with their provider about their journal information and it helped Ps feel more prepared for their visit (60%) and provide more information to provider (53%).

References

25. Shimada SL, Allison JJ, Rosen AK, Feng H, Houston TK. Sustained Use of Patient Portal Features and Improvements in Diabetes Physiological Measures. J Med Internet Res 2016;18(7):e179. doi:10.2196/jmir.5663. PMID: 27369696
26. Ronda MC, Dijkhorst-Oei L-T, Rutten GE. Patients' experiences with and attitudes towards a diabetes patient web portal. PLoS One 2015;10(6):e0129403. doi:10.1371/journal.pone.0129403. PMID: 26086272
27. Ronda MC, Dijkhorst-Oei L-T, Rutten GE. Reasons and barriers for using a patient portal: Survey among patients with diabetes mellitus. J Med Internet Res 2014;16(11):e263. doi:10.2196/jmir.3457. PMID: 25424228
28. Roelofsen Y, Hendriks SH, Sieverink F, Landman GW, Groenier KH, Bilo HJ, Kleefstra N.. Differences between patients with type 2 diabetes mellitus interested and uninterested in the use of a patient platform (e-VitaDM-2/ZODIAC-41). J Diabetes Sci Technol 2014;8(2):230-237. doi:10.1177/1932296814524496. PMID: 24876572
29. Sarkar U, Lyles CR, Parker MM, Allen J, Nguyen R, Moffet HH, Schillinger D, Karter AJ. Use of the refill function through an online patient portal is associated with improved adherence to statins in an integrated health system. Med Care 2014;52(3):194-201. doi:10.1097/MLR.0000000000000069. PMID: 24374412
30. Sieverink F, Kelders SM, Braakman-Jansen LMA, Van Gemert-Pijnen JEWC. The added value of log file analyses of the use of a personal health record for patients with type 2 diabetes mellitus: Preliminary Results. J Diabetes Sci Technol 2014;8(2):247-255. doi:10.1177/1932296814525696. PMID: 24876574
31. Lyles CR, Sarkar U, Ralston JD, Adler N, Schillinger D, Moffet HH, Huang ES, Karter AJ. Patient-provider communication and trust in relation to use of an online patient portal among diabetes patients: The Diabetes and Aging Study. J Am Med Inform Assoc 2013;20(6):1128-1131. doi:10.1136/amiajnl-2012-001567. PMID: 23676243
32. Ronda MC, Dijkhorst-Oei LT, Gorter KJ, Beulens JW, Rutten GE. Differences between diabetes patients who are interested or not in the use of a patient Web portal. Diabetes Technol Ther 2013;15(7):556-563. doi:10.1089/dia.2013.0023. PMID: 23777369
33. Tenforde M, Nowacki A, Jain A, Hickner J. The association between personal health record use and diabetes quality measures. J Gen Intern Med 2011;27(4):420-424. doi:10.1007/s11606-011-1889-0. PMID: 22005937
34. Bredfeldt CE, Compton-Phillips AL, Snyder MH. Effects of between visit physician-patient communication on Diabetes Recognition Program scores. Int J Qual Heal Care 2011;23(6):664-673. doi:10.1093/intqhc/mzr061. PMID: 21937586
35. Sarkar U, Karter AJ, Liu JY, Adler NE, Nguyen R, López A, Schillinger D. Social disparities in internet patient portal use in diabetes: evidence that the digital divide extends beyond access. J Am Med Inform Assoc 2011;18(3):318-321. doi:10.1136/jamia.2010.006015. PMID: 21262921
36. Cho AH, Edelman DE, Hartwell PH, Oddone EZ, Yancy WS, Carolina N. Do Diabetic Veterans Use the Internet? Self-Reported Usage, Skills, and Interest in Using My HealtheVet Web Portal. Telemed J E Health 2010;16(5):595-602.
37. Sarkar U, Karter AJ, Liu JY, Adler NE, Nguyen R, Lopez A, Schillinger D. The literacy divide: Health literacy and the use of an internet-based patient portal in an integrated health system-results from the diabetes study of northern California (DISTANCE). J Health Commun 2010;15 Suppl 2(March 2016):183-196. doi:10.1080/10810730.2010.499988. PMID: 20845203

38. Weppner WG, Ralston JD, Koepsell TD, Grothaus LC, Reid RJ, Jordan L, Larson EB. Use of a shared medical record with secure messaging by older patients with diabetes. Diabetes Care 2010;33:2314-2319. doi: 10.2337/dc10-1124. PMID: 20739686
39. Harris LT, Haneuse SJ, Martin DP, Ralston JD. Diabetes quality of care and outpatient utilization associated with electronic patient-provider messaging: A cross-sectional analysis. Diabetes Care 2009;32(7):1182-1187. doi:10.2337/dc08-1771. PMID: 19366959
40. Wald JS, Grant RW, Schnipper JL, Gandhi TK, Poon EG, Businger AC, Orav EJ, Williams DH, Volk LA, Middleton B. Survey analysis of patient experience using a practice-linked PHR for type 2 diabetes mellitus. AMIA Annu Symp Proc 2009;2009:678-682. PMID: 20351940
